# Joint torque and electromyographic activity during eccentric exercise for hip adductors at different hip flexion angles

**DOI:** 10.1371/journal.pone.0336699

**Published:** 2025-11-12

**Authors:** Jan Marušič, Oskar Cvjetičanin, Nejc Šarabon

**Affiliations:** 1 Faculty of Health Sciences, University of Primorska, Koper, Slovenia; 2 Ludwig Boltzmann Institute for Rehabilitation Research, Sankt Pölten, Austria; 3 Innorenew CoE, Izola, Slovenia; Faculty of Health Sciences, University of Primorska, SLOVENIA

## Abstract

Our objective was to investigate the effects of three hip flexion angles (0°, 45°, and 90°) and leg dominance on peak joint torque, angle at peak torque, and peak electromyographic activity (EMGA) of the adductor longus during bilateral eccentric hip adduction. Sixteen recreationally active participants completed bilateral eccentric contractions at each hip flexion angle using a custom-built dynamometer. The primary outcome measures were peak adduction torque, angle at peak torque, and EMGA of the adductor longus. A 3 × 2 repeated measures ANOVA was used to assess the effects of hip angle and leg dominance. Hip flexion angle had a significant main effect on peak torque (F(2, 30) = 15.75, p < 0.01), with peak torque significantly lower at 90° compared to 0° and 45°. No significant effects were observed for leg dominance or interaction. No significant main effects of hip flexion angle, leg dominance, or their interaction were observed on peak EMGA or peak torque angle. Eccentric hip adduction strength is reduced at 90° of flexion, likely due to mechanical disadvantage, while neural activation remains unchanged. These findings support the use of less flexed positions in eccentric training protocols for adductor strength development or injury prevention.

## Introduction

Groin injuries present a significant problem in sport due to their high injury rate, long-lasting symptoms and high risk of re-injury [1]. Hip adductor strains are the most common subtype, which accounts for approximately two-thirds of all groin injuries, with the adductor longus being the most commonly injured [2–5]. The main risk factors are previous injury and reduced hip adductor strength [6]. These injuries often result in diminished performance and significant loss of time. For instance, prospectively collected data from professional football players across two seasons reported a median time loss of 10 days per groin injury (interquartile range: 5–22 days) [4]. Therefore, implementing effective prevention strategies—particularly those aimed at improving hip adductor strength—is essential.

However, the effectiveness of injury prevention programmes targeting groin injuries has often been limited, with many failing to demonstrate statistically significant effects [7,8]. In preventing hamstring injuries, eccentric exercises such as the Nordic hamstring exercise have proven highly effective [9,10]. The benefits of such training are thought to result from a range of neuromuscular and structural adaptations, including increased muscle fascicle length, enhanced maximal eccentric strength, improved flexibility, a shift in the angle of peak torque toward longer muscle–tendon lengths, and increased maximal voluntary neuromuscular activation [11–14]. Considering that hamstring strains share a similar injury mechanism to adductor strains (i.e., rapid activation during lengthening of the muscle-tendon complex for the purpose of decelerating the lower limb) [15], it is plausible that eccentric training could offer similar protective benefits for the hip adductors.

Several studies have evaluated various hip adductor strengthening exercises [16–18] and clinical examination isometric tests [19–22] by measuring joint torque and/or electromyographic muscle activity (EMGA) of the adductor longus to facilitate the design of training programmes, the assessment of strength and the detection of groin injury. However, none have examined eccentric exercises with supramaximal loading for the hip adductors. Addressing this gap is critical, as simultaneous assessment of EMGA and joint torque offers insight into both neural activation and mechanical output—an especially relevant consideration during eccentric loading, where these parameters can diverge. Such a dual perspective enables a more comprehensive understanding of neuromuscular efficiency, informs risk assessment for injury, and guides the optimisation of exercise selection.

Moreover, performing exercises for hip adductors in a neutral hip position in the sagittal plane may not be biomechanically optimal for strength development or injury prevention. Indeed, one of the lesser known and researched aspects of strength training for the hip adductors is the influence of the hip flexion angle during hip adductor contractions. Studies have demonstrated that during maximal isometric contractions of hip adductors, peak hip adductor EMGA and peak joint torque vary depending on the hip flexion angle [19–22]. Specifically, participants attained maximum torque and EMGA of the hip adductors at 0° or 45° of hip flexion, while values were significantly reduced at 90° [19,21]. Whether these findings extend to eccentric loading remains unknown.

Understanding how hip flexion angle influences EMGA and torque in eccentric hip adductor exercises is key for effective strength training, injury prevention or rehabilitation. This study aims to investigate the effects of 0°, 45°, and 90° hip flexion on peak torque and EMGA of the adductor longus using a custom-made isokinetic dynamometer. We hypothesise that 90° flexion would elicit significantly lower peak torque and EMGA than 0° and 45°.

## Materials and methods

### Participants

The minimum acceptable sample size was calculated using the G*Power software [23]. The calculation indicated that 16 participants were required, assuming a medium effect size (f = 0.25), a statistical power of 80%, and a significance level of α = 0.05 for a repeated-measures ANOVA. Sixteen recreationally active participants participated in this study: 11 men (age: 28.2 ± 4.4 years, body height: 1.85 ± 0.05 m, body weight: 83.0 ± 11.6 kg, body mass index: 24.1 ± 2.7 kg/m^2^) and 5 women (age: 25.4 ± 4.6 years, body height: 1.67 ± 0.04 m, body weight: 61.4 ± 5.5 kg, body mass index: 22.1 ± 1.4 kg/m^2^). The inclusion criteria required the absence of any musculoskeletal injuries or pain syndromes in the past year, performing regular physical activity and having experience with strength training. Recruitment was conducted between September 1, 2023, and October 31, 2023. Before starting the measurements, participants were informed in detail about the protocol and had to sign a written informed consent form. The protocol was conducted in accordance with the latest revision of the Declaration of Helsinki. The study was conducted in accordance with the latest revision of the Declaration of Helsinki. The experimental procedures were reviewed and approved by the National Medical Ethics Committee of the Republic of Slovenia (reference number: 0120–690/2017/8).

### Study design and procedures

This cross-sectional study involved bilateral eccentric hip adductor exercises on a custom-built isokinetic dynamometer after a standardised warm-up. Participants were familiarised with the device beforehand. The warm-up included 6 min of step-ups on a 25 cm box, dynamic stretching and strengthening (hip circles, flexions, squats, lunges, bridges, sit-ups), and graded eccentric contractions at 25%, 50%, 75% and 90% of maximal voluntary contraction. Range of motion was set individually to a slight, pain-free stretch. After warm-up, wireless EMG electrodes (Trigno Delsys, USA) were placed on the adductor longus of both legs, positioned one-third along the medial thigh from the pubic symphysis to the medial femoral condyle [24]. Prior to electrode placement, each participant’s skin was prepared according to SENIAM guidelines to reduce impedance. This involved shaving the skin, lightly abrading it and then wiping the electrode site with an alcohol wipe. To confirm the correct placement of the electrodes, a manual muscle test was performed and the EMGA signal was checked. Participants then performed two 5-s maximal voluntary isometric bilateral contractions of hip adductors in the supine position with the hip and knee in neutral position and a ball placed between the knees (centered on the medial epicondyles of femur) for the purpose of EMGA normalization [16,18].

**Fig 1:**
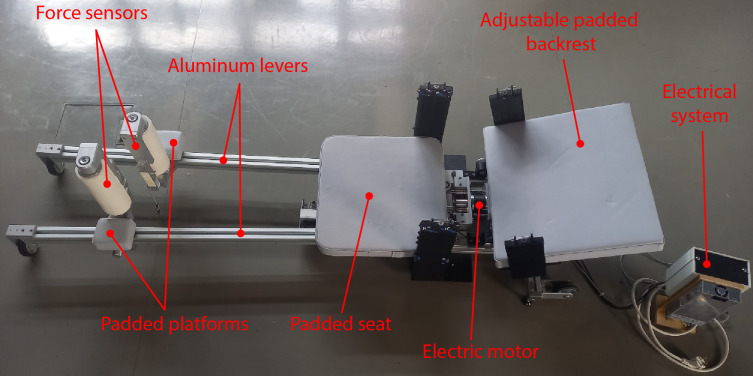
A custom-built isokinetic dynamometer that enables eccentric contractions of the hip adductors.

**Fig 2:**
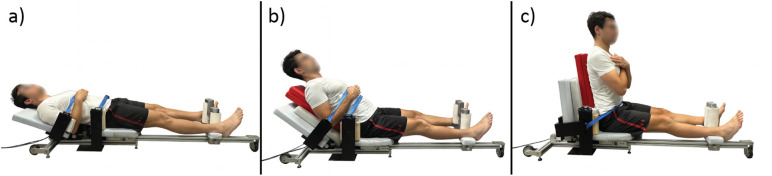
The positioning of the participant at 0° of hip flexion (a), 45° of hip flexion (b), and 90° of hip flexion (c).

Custom-built dynamometer was designed to allow bilateral isokinetic eccentric contractions of the hip adductors at a fixed angular velocity of 13°/s, across various hip flexion angles (Fig 1). This velocity allows most participants to reach the end range of motion in hip abduction within approximately three seconds of eccentric loading. The design of the dynamometer is similar to conventional gym training equipment designed for strengthening the hip adductors in a seated position. The user can either sit or lie on the device as the adjustable backrest allows for angles between 0° and 90° (Fig 2). Users’ legs are resting on the padded platforms connected to aluminium levers and are moved apart by a 24V DC electric motor, forcing the individual into hip abduction. Two force sensors (PW2DC3, HBK, Darmstadt, Germany) are installed on padded platforms to measure force exerted by each leg during the isokinetic movement. These sensors have shown excellent reliability in similar dynamometers used for isometric knee strength testing [25]. The platforms can slide along aluminium levers, allowing for individual adjustment to accommodate users of different sizes. The electric motor is powered and controlled by a custom-designed electrical system, which makes the device easy to operate. The abduction angle of the hip is measured by the change in the displacement of the padded platforms relative to the starting position (via a linear transducer SX80, WayCon, Munich, Germany). Preliminary analyses from an additional, yet unpublished study demonstrated excellent reliability of the described dynamometer (intraclass correlation coefficient > 0.9, coefficient of variation < 10%).

Three variations of bilateral eccentric hip adduction (hip flexion at 0°, 45°, and 90°) were performed in random order, five repetitions each, with five minutes rest. Hip flexion angles were set via the dynamometer’s adjustable backrest. Contact with the dynamometer was just above the medial malleolus, and the distance to the greater trochanter (hip axis) was measured to calculate joint torque. Participants were positioned so the greater trochanter aligned with the dynamometer’s axis, secured with pelvic and chest straps, and instructed to resist maximally through the full range as the device moved their legs into abduction.

The force and angle signals from the dynamometer were acquired at sampling frequency of 1000 Hz and processed using dedicated ARS software (S2P, Science to Practice, Ltd., Ljubljana, Slovenia). The signals were low-pass filtered using a fourth-order Butterworth filter with a cutoff frequency of 20 Hz. For each repetition, the highest mean forces were determined for each leg in a 0.1-s window. The forces were then multiplied by the lever distance, resulting in the unilateral peak hip joint torque [Nm] of the best (highest peak torque) out of five repetitions for each exercise variation. Full range of motion and hip angle at the moment of peak torque were also obtained. The EMGA data were acquired at 1000 Hz using the Trigno wireless system and its corresponding software (Delsys Inc., USA). Data were processed in the following order: 1) band pass filtration using Butterworth second-order filter (20–500 Hz), 2) rectification, using root mean square function (0.05-s window length and point-by-point overlap), 3) smoothing, using moving average function (0.05-s window length and point-by-point overlap). The main outcome measure was the peak EMGA, which was determined as the highest mean value at a 0.25-s window length and expressed as percentage of the peak EMGA during maximal isometric trials.

### Statistical analysis

All statistical analyses were conducted using SPSS Statistics software (version 26.0, IBM: Armonk, NY, USA). Descriptive statistics were calculated and reported as mean ± standard deviation. The normality of the data distributions for all variables was verified with the Shapiro–Wilk test. A two-way repeated measures analysis of variance (3 × 2) was performed to evaluate the main effects and interaction of hip flexion angle (0°, 45°, and 90°) and leg dominance (dominant vs. non-dominant) on the outcome variables. Mauchly’s test of sphericity was used to test the sphericity assumption, and if violated, the Greenhouse–Geisser correction was applied. Partial eta squared (η^2^) effect sizes were calculated and interpreted as small (0.01–0.06), medium (0.06–0.13) and large (> 0.14). When significant main effects were found, Bonferroni-adjusted paired t-tests were used for post hoc comparisons. Cohen’s d effect sizes were calculated and interpreted as trivial (< 0.2), small (0.2–0.5), medium (0.5–0.8), large (0.8–1.2), very large (1.2–2.0), and huge (> 2.0) [26]. The level of statistical significance was set at α = 0.05. Due to poor EMGA signal quality in two participants—identified through visual inspection as showing non-physiological, artefactual activity—the EMGA data from these individuals were excluded. Consequently, statistical analyses for EMGA were conducted on a reduced sample of 14 participants.

## Results

**Fig 3:**
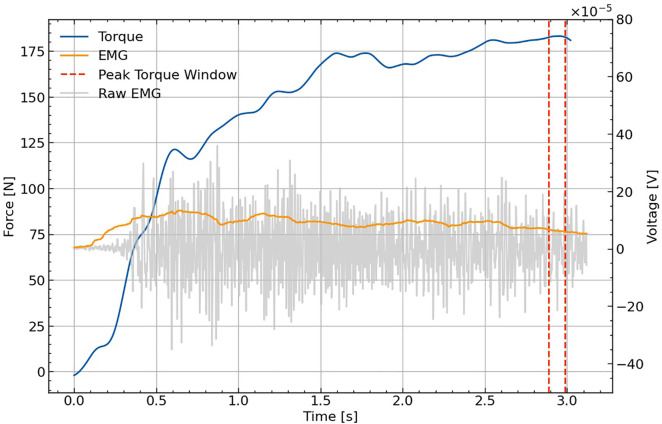
An example of the torque (blue line), raw electromyographic signal (grey line) and processed electromyographic signal (yellow line) of adductor longus during one repetition. Two dotted lines indicate the position of the peak torque (highest mean value at a window length of 0.1 **s)**.

All participants successfully performed all variations of the eccentric exercise for hip adductors on the dynamometer. The mean range of motion for all three variations was 42.7° ± 13.5°. A representative example of the torque and joint angle signals for one repetition is shown in Fig 3.

### Descriptive statistics

The descriptive statistic of outcomes across three different hip flexion angles (0°, 45°, and 90°) are shown in Table 1.

**Table 1:** Descriptive statistics (mean ± standard deviation).

	Hip 0°		Hip 45°		Hip 90°	
	DL	NDL	DL	NDL	DL	NDL
**Peak torque [Nm]**	217.3 ± 57.2	208.8 ± 50.5	208.7 ± 64.1	198.6 ± 58.9	177.0 ± 57.4	170.7 ± 59.2
**Angle at** **peak torque [°]**	34.6 ± 12.5	36.0 ± 13.0	35.4 ± 13.1	36.2 ± 13.4	37.0 ± 13.6	34.7 ± 13.7
**EMGA of AL [%]**	0.97 ± 0.34	1.08 ± 0.39	1.01 ± 0.42	0.90 ± 0.33	0.92 ± 0.38	0.92 ± 0.30

DL dominant leg, NDL non-dominant leg, EMGA electromyographic activity, AL adductor longus

### Effects of hip flexion (0°, 45°, and 90°) and leg (dominant vs. non-dominant) on peak torque

A two-way repeated measures analysis of variance revealed a significant main effect of hip flexion on peak torque (F(2, 30) = 15.75, p < 0.01, η² = 51).

A further analysis was performed to compare the three exercises for each leg separately with 1-way repeated measures ANOVA. There were significant differences between exercises for the dominant leg (F(2, 30) = 15.53, p < 0.01, η² = 0.51) and for the non-dominant leg (F(2, 30) = 14.51, p < 0.01, η² = 0.49). Pairwise comparisons showed for both legs that peak joint torque was significantly lower during the variation with 90° hip flexion compared to both 0° (dominant leg: p < 0.01, d = 0.70; non-dominant leg: p < 0.01, d = 0.70) and 45° variations (dominant leg: p < 0.01, d = 0.52; non-dominant leg: p < 0.01, d = 0.47) while there were no significant differences between the 0° and 45° variations (p = 0.29–0.79).

There was no significant main effect of leg, F(1, 15) = 3.82, p = 0.07, and no significant interaction between hip flexion angle and leg, F(2, 30) = 0.69, p = 0.51.

### Effects of hip flexion (0°, 45°, and 90°) and leg (dominant vs. non-dominant) on angle at peak torque

A two-way repeated measures analysis of variance revealed no significant main effect of hip flexion angle on angle of peak torque (F(2, 30) = 0.10, p = 0.90), no significant main effect of leg on angle of peak torque (F(1, 15) = 0.00, p = 0.97), and no significant interaction between hip flexion angle and leg (F(1.2, 17.9) = 3.36, p = 0.07).

### Effects of hip flexion (0°, 45°, and 90°) and leg (dominant vs. non-dominant) on EMGA of adductor longus

A two-way repeated measures analysis of variance revealed no significant main effect of hip flexion angle on peak adductor longus EMGA (F(2, 30) = 1.34, p = 0.28), no significant main effect of leg on peak adductor longus EMGA (F(1, 15) = 1.85, p = 0.20), and no significant interaction between hip flexion angle and leg on peak adductor longus EMGA (F(1.4, 18.4) = 1.97, p = 0.18).

## Discussion

This is the first study to examine the effects of hip flexion angle (0°, 45°, 90°) and leg dominance on peak torque and EMGA of the adductor longus during slow bilateral eccentric contractions. Hip flexion angle significantly affected peak torque, which was lower at 90° than at 0° and 45°, but did not influence EMGA.

The reason why the maximum eccentric hip adduction strength is reduced at extreme hip flexion angles around 90° can probably be explained by the synergistic roles of the hip adductors. Up to an angle at approximately 90° (when the insertion on the femur becomes superior to the origin of a muscle), they contribute significantly to hip flexion; beyond this point, they may act more as hip extensors [27]. This could indicate that at 90° of hip flexion there is a suboptimal force-length relationship and decreased mechanical advantage (e.g., moment arm and line of pull) of the hip adductors. This trend was also found in two studies in which isometric hip adduction strength was measured at different hip flexion angles [19,21]. Concerning these findings, it should be noted that conventional exercise machines in commercial gyms designed to strengthen the hip adductors often limits performance to a seated position (i.e., at 90° of hip flexion). While this type of design provides convenience and less space consumption, it may not provide the optimal training stimulus according to the results of our study. Furthermore, it can be surmised that the seated position is the least likely to adequately mimic the dynamic demands during the critical sporting activities where most hip adductor injuries occur (kicking the ball, rapid changes of direction, reaching, jumping) [15].

In addition, the results of studies comparing 0° and 45° of hip flexion are rather contradictory. In our study, there were no significant differences in peak torque; in the study by Delahunt et al. [21] isometric peak torque was significantly higher at 45°, while the opposite was true in the study by Lovell et al. [19]. Differences in methodology and participant characteristics could partly explain these results, as Delahunt et al. [21] examined male Gaelic games players with an average age of 21 years and measured pressure values with a sphygmomanometer, whereas Lovell et al. [19] tested junior soccer players with an average age of 16 years using load cells. Such differences in measurement equipment, type of contraction, and in participants’ age, training experience, and sport-specific demands could therefore contribute to the observed discrepancies in torque production between studies. Furthermore, in the study by Light et al. [20], the participants also generated a significantly higher peak torque (+ 69%) at 0° compared to 45° hip flexion. However, their isometric measurements at 0° of hip flexion were performed at a significantly higher hip abduction angle, as the nature of the test required the tester to place the entire forearm (26.5 cm long) between the legs in addition to the hand-held dynamometer. The fact that higher hip abduction angles represent a mechanically more favourable position for hip adductor torque production was also demonstrated by Welsh et al. [28], as participants achieved significantly higher peak isometric torques at 10° and 20° of hip abduction than in the neutral position at 0°. This trend is consistent with our results, as participants achieved their peak hip torque towards the end of the range of motion regardless of hip flexion angle (mean angle at peak torque was 35–37° and mean range of motion was 42.7°). Similar peak torque angles were observed during concentric isokinetic measurements (20°/s) in the study by Menegaldo et al. [29].

Interestingly, the hip flexion angle influenced the peak torque but not the peak EMGA of the adductor longus. This finding suggests that while mechanical output (joint torque) varies with hip flexion angle, the neural drive to the adductor longus remains relatively constant across the tested positions. One possible explanation is that other hip adductors may contribute differently to total torque at varying hip flexion angles, compensating for changes in mechanical advantage. Alternatively, it is also plausible that muscle length and joint angle influence the force-generating capacity of the muscle through alterations in sarcomere overlap, moment arm geometry, and passive tension, without necessarily altering the level of EMGA. Our findings are in contrast to the results of studies on EMGA during isometric contractions at different hip flexion angles, which all reported significantly higher EMGA at 45° compared to 0 and 90° [16,18,19]. The reason for these discrepancies could be the type of contraction, as it is known that EMGA amplitudes can behave differently during eccentric contractions. According to Enoka [30], the neural commands during eccentric contractions may alter the order of recruitment, threshold and discharge rate of motor units, and their EMGA amplitudes are generally lower due to greater metabolic efficiency. Additionally, trunk and pelvic stabilization demands during eccentric loading through a full range of motion may increase compared with isometric exercises, which could in turn influence hip adductor EMG patterns. In any case, we must be aware that the EMGA measurement in our study only provides a small insight into a part of one of the five muscles classified as hip adductors, and that some degree of crosstalk from adjacent muscles in this region may also have influenced the recorded signal. Nevertheless, our results provide important new information about the EMGA of the adductor longus during supramaximal eccentric bilateral exercises with different hip angles. Several studies have investigated peak EMGA values during various hip adductor exercises, but only under isometric or concentric-eccentric conditions [16–19,21,22]. The peak EMGA values of 90.4–104.8% during our supramaximal eccentric bilateral contractions are comparable to the peak EMGA values of dynamic (non-isometric) exercises that elicited the highest EMGA of the adductor longus (99–108% during the Copenhagen adduction exercise, standing hip adduction with elastic band, sliding hip adduction/abduction and hip adduction on the machine (see Serner et al. [16] for a description of the exercises)). Despite the similar peak EMGA, the peak torques in supramaximal bilateral eccentric contractions are likely to be much higher than in the concentric-eccentric exercises mentioned above. In the long term, this type of eccentric training could be more effective in improving maximal eccentric strength and therefore exert a greater positive impact on the greatest modifiable risk factor for groin injury – low hip adductor strength. In addition to strength gains, eccentric training may also induce beneficial morphological and functional adaptations, such as increased muscle fascicle length and enhanced flexibility [12]. However, these are hypotheses that have not (yet) been verified and would be worth investigating in further studies.

Some limitations should be noted. The study involved a heterogeneous group of moderately trained individuals, so findings may not generalise to highly trained athletes with sport-specific demands that chronically load the hip adductors. EMGA of the adductor longus is technically challenging, and electrode shift or crosstalk from nearby muscles cannot be fully excluded, particularly in the maximally stretched position. In addition, the use of a bilateral contraction protocol does not necessarily reflect the outcomes that might be obtained from unilateral testing, which could more specifically represent the unilateral loading patterns typically observed during natural movements or sport-specific tasks. Furthermore, the custom-made dynamometer, despite its excellent reliability, may limit the direct comparability of the obtained results due to the lack of validation against a standard isokinetic dynamometer, which is also restricted to unilateral testing.

## Conclusions

This study demonstrates that hip flexion angle substantially influences peak eccentric hip adduction torque, with strength notably reduced at 90° compared to 0° and 45°, while neural activation of the adductor longus remains unaffected. The reduction at higher flexion angles is likely due to a less favourable force–length relationship and diminished mechanical advantage. These results underline the importance of considering hip position in both testing and training contexts. Performing eccentric strengthening in more extended hip positions (0° or 45°) may offer a greater mechanical stimulus and better reflect the demands of sport-specific movements.
